# Couple-Years of Protection Indicator: New Global Guidance for Updating Existing Methods and Adding New Methods

**DOI:** 10.9745/GHSP-D-23-00388

**Published:** 2024-04-29

**Authors:** Elena Lebetkin, Markus J. Steiner, Emily Sonneveldt, Amani Selim, Bamikale Feyisetan, Baker Maggwa Ndugga, A. Wezi Munthali, Morrisa Malkin, Fatou Jallow

**Affiliations:** a FHI 360, Durham, NC, USA.; b Avenir Health, Glastonbury, CT, USA.; c Public Health Institute Contractors with USAID Global Health Training, Advisory and Support Contract, Washington, DC, USA.; d National Cancer Institute, National Institutes of Health, Bethesda, MD, USA.

## Abstract

**Background:**

Couple-years of protection (CYP) is an indicator that allows for monitoring and evaluating of family planning (FP) program performance through simple calculations. The CYP for each contraceptive method is calculated by multiplying the number of contraceptive commodity units distributed to clients over a 1-year period by a conversion factor that quantifies the duration of contraceptive protection provided per unit distributed. CYP calculations across methods were previously updated in 2000 and 2011, resulting in changes in methodology, factor inclusion, and specific methods. Since the 2011 update, changes and additions to the modern contraceptive method mix required new CYP conversion factors for 4 methods of contraception: Levoplant implant, progestin-only pills (POPs), Caya diaphragm, and the hormonal intrauterine device.

**Methods:**

We conducted literature reviews of both published and gray literature and consulted with experts to identify updated data on continuation rates, duration of efficacy, and method effectiveness for the 4 methods. New CYP conversion factors were calculated for the 4 methods either by using the same calculation used previously for the method considering new data or, for new methods, using calculations for similar methods.

**Results:**

New CYP conversion factors were assigned to the 4 methods of contraception covered in this update: Levoplant, 2.5 CYP per implant inserted; POPs, 0.0833 CYP per pack (i.e., 12 cycles per CYP); Caya diaphragm, 1 CYP per device, and hormonal intrauterine device, 4.8 CYP per device inserted.

**Conclusions:**

CYP is an important indicator for FP programs. As new methods of contraception are developed and new evidence is generated for current methods, the indicator may need to be updated. A standard process for updating and documenting future CYP updates is recommended.

## Abstract (precis)

Couple-years of protection is an important indicator for measuring coverage of family planning programs. Method-specific updates are required when a new method is introduced, a regulatory body changes the duration of use, or a significant change in presentation occurs.

## Key Messages

- We describe our approach to the most recent update of the couple-years of protection (CYP) indicator for 4 contraceptive methods. Program implementers can use the updated indicator for improved program planning, monitoring, and evaluation.
- We present a flowchart to determine when and whether to update CYP conversion factors and to offer a standardized process for future CYP updates.
- Policymakers and program-level decision-makers can use the new CYP data to monitor family planning program performance, identify gaps in contraceptive service delivery, and devise approaches to improve equitable access to family planning.

## BACKGROUND

Family planning (FP) programs rely on effective indicators to assess performance, measure progress, and inform data-driven decision-making. Couple-years of protection (CYP) is an output indicator widely used by international organizations, governments, and donors to track program performance and estimate the FP method protection “coverage*” required to meet the contraceptive needs of the populations they serve.

The introduction of this indicator in 1973^1^ marked an important shift in how FP programs were measured and evaluated. Previously, programs depended on the number of acceptors, regardless of the method. The limitation of this approach is that contraceptive methods vary in the level of protection they offer. For example, a couple accepting a supply of condoms may receive limited pregnancy protection for a short period of time, while a couple choosing sterilization will benefit from near-perfect pregnancy protection until menopause. With the introduction of the CYP indicator, however, programs were able to calculate the protection from pregnancy afforded by a specific method or sum all method-specific CYPs to obtain an aggregate total figure. The reason the CYP indicator was so revolutionary for FP programs lies in its simplicity: programs can use readily available data, such as service statistics or data from the health management information system, and simply multiply the number of units distributed over a 1-year period by a conversion factor that quantifies the duration of contraceptive protection provided per unit distributed. Depending on the method, the conversion factor relies on different inputs, such as coital frequency, method effectiveness, and duration of use.^1^^–^^5^ As detailed by Stover et al.,^4^ the conversion factor does not take the reason for use into account; for example, condoms used for HIV prevention are assigned the same conversion factor as condoms used for contraception. Along with other commonly used FP indicators, such as contraceptive prevalence rate,^2^ CYP can help program managers, governments, donors, and other entities obtain a comprehensive picture of program performance.

As new methods or new data on existing methods become available, the CYP indicator must be updated to ensure it reflects the current landscape of contraception options and enables accurate monitoring and evaluation of FP programs. The indicator has been updated 3 times since its inception: in 2000, by the U.S. Agency for International Development (USAID)-funded EVALUATION Project^4^; in 2011, by the USAID-funded RESPOND Project^5^; and in 2022, by FHI 360, Avenir Health, and USAID^3^ (Box). Due to the COVID-19 pandemic, we were unable to hold a consensus meeting to validate the new CYP factors. However, a series of virtual meetings to gain consensus were held in 2021 and 2022 with a wide variety of international FP stakeholders representing donor organizations, implementing partners, and academic institutions. For additional information on this and previous updates, refer to the report that provides a more detailed explanation.^3^ In this article, we discuss the 2022 updates and propose a standardized process for future CYP updates.

BOX Summary of Couple-Years of Protection Updates Conducted in 2000 and 2011

**2000 Update Highlights**

- Country or regional conversion factors for sterilization were added as the age at sterilization varies substantially across contexts.
- Couple-years of protection (CYP) figures for condoms and spermicide were reduced due to new data from the Demographic and Health Surveys, which showed a lower coital frequency than previously estimated.

**2011 Update Highlights**

*Evidence Considered in CYP Calculations*

- U.S.-based clinical trials would no longer be used to estimate contraceptive continuation rates as these rates are typically higher than continuation rates from studies in low- and middle-income countries.
- Studies with specific/special populations, such as women using barrier methods primarily for sexually transmitted infection prevention, would no longer be considered for inclusion in the evidence to estimate CYP factors.

*CYP Calculation Changes*

- Final CYP factors will no longer be rounded to the nearest whole number.
- Overlapping coverage—defined as use of a method while the woman is less than 6 months postpartum, currently amenorrheic, and breastfeeding, or when more than 1 method of contraception is used—will no longer be included in the calculation, as a very small percentage of women fall into this category.
- Method effectiveness will no longer be included as a separate component in the CYP calculation for long-acting reversible contraception, as effectiveness is already accounted for in method continuation data.

*Methods With Updated CYP*

- IUDs, implants, sterilization, and fertility awareness methods had new CYPs calculated.
- Diaphragm was dropped as the product was marginally used.
- New methods—vaginal ring and hormonal patch—were added.
- Levoplant (formerly called Sino-Implant (II)) was added post-2011 with a 4-year duration of efficacy.

The CYP indicator must be updated to ensure it reflects the current landscape of contraception options and enables accurate monitoring and evaluation of FP programs.

## STANDARDIZED PROCESS FOR COUPLE-YEARS OF PROTECTION UPDATES

Since the advent of the indicator, CYP updates have occurred as needed at USAID’s request, and documentation of the process and methods used to conduct the updates has varied. We reviewed the methods used and documentation available for previous CYP updates.^4^^,^^5^ In an effort to streamline and standardize the process moving forward, we determined that processes used in past CYP updates should be adhered to as closely as possible and methodological assumptions should not be changed without strong justification. As such, the CYP update outlined in this article closely follows prior processes. We also propose a standard process for determining if a CYP update is needed in the future and, if so, how to calculate updated CYP factors.

The Figure outlines the following 3 criteria that should trigger a CYP update and provides a simple flowchart to guide the decision-making process. Once the decision is made that a method needs an updated CYP factor, we recommend following the process outlined in the methods section to calculate the updated CYP factor.

**FIGURE:**
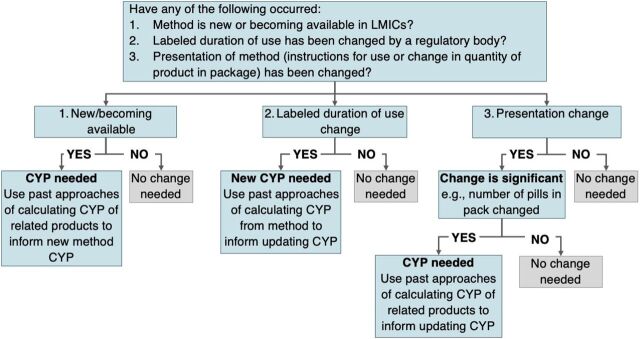
Flowchart to Determine if Couple-Years of Protection Update Is Needed Abbreviations: CYP, couple-years of protection; LMIC, low- and middle-income country.

### 1. A Contraceptive Method Is New or Becoming Available in Low- and Middle-Income Countries

New methods do not have a CYP assigned and, thus, need a CYP for use moving forward. A new method of contraception is one that recently received regulatory approval and is now being distributed for use in the general population. A newly available method is one that has been available in select countries (usually higher-income countries) for any period of time and has recently become available in low- and middle-income countries (LMICs) for a number of possible reasons, including manufacturers offering the product at a more affordable price, regulatory approval by a Stringent Regulatory Authority or prequalification by the World Health Organization (WHO) making the product available for order by funders, social marketing organizations introducing the product in country, and/or significant pilot projects by organizations that have led to the product being available in LMICs.

### 2. The Labeled Duration of Use for a Method Has Been Changed by a Regulatory Body

At times, due to new evidence, a regulatory body may change the labeled duration of use of a method that necessitates a change in CYP for the method. For example, as discussed in this article, the hormonal intrauterine device (IUD) was changed from a 5- to a 7-year method by the WHO and U.S. Food and Drug Administration (FDA) in 2019 and subsequently to an 8-year method by the FDA in 2022.

### 3. The Method Presentation Has Changed Significantly

A significant change in the method presentation is defined as a change that affects the CYP calculation and is a scenario that was not present in previous CYP updates. For example, as outlined in this update, the progestin-only pill (POP) blister pack procured by USAID now consists of 35 pills rather than the standard 28 pills.

## RATIONALE FOR CURRENT COUPLE-YEARS OF PROTECTION UPDATE

Since 2011, notable developments in the modern contraceptive method mix led the CYP working group in the USAID Office of Population and Reproductive Health to recommend a CYP update for 4 methods: Caya diaphragm, Levoplant, the hormonal IUD, and the POP blister pack of 35 pills (Table). These methods were selected based on their alignment with the 3 primary reasons for CYP update previously listed: (1) Caya diaphragm is newly available in LMICs^6^; (2) A regulatory body changed the duration of efficacy for Levoplant (from 4 to 3 years) and the hormonal IUD (from 5 to 7 years); and (3) The presentation of POP blister packs changed from 28 pills to 35 pills per pack (for USAID-procured packs). The guidance outlined in this article is intended for USAID-funded programming and, therefore, applies to USAID-procured commodities. However, it is important to note the only commodity discussed in this article that is specific to USAID programs is the POP blister pack of 35 pills. Based on global procurement data since 2017,^7^ Zimbabwe and Eswatini are the only countries that ordered and/or received the 28-day cycle POP presentation, and these commodities were procured by UNFPA. Thus, USAID-procured POPs are in line with the vast majority of POPs procured globally.

**TABLE.** Updated Couple-Years of Protection Indicator

**Contraceptive Method**	**CYP Per Unit**
Diaphragm	1 per diaphragm
Levoplant	2.5 per implant
Hormonal intrauterine device	4.8 per intrauterine device
Progestin-only pills blister pack of 35 pills	0.0833 per pack (12 cycles per CYP)

Notable developments since 2011 in the modern contraceptive method mix led the CYP working group to recommend a CYP update for the Caya diaphragm, Levoplant, hormonal IUD, and the POP blister pack of 35 pills.

## METHODS

First, a desk review of studies published since 2011 (the most recent CYP update) on the 4 methods was conducted. Studies that have data for the factors included in CYP calculations were focused on method effectiveness, duration of use, coital frequency, consistency of use, and wastage. Consistent with the 2011 recommendations, we excluded U.S.-based clinical trials, studies on specific/special populations, and literature on overlapping coverage. We then reviewed the references of published studies and contacted subject matter experts and manufacturers to identify any additional published, unpublished, and gray literature. We then synthesized the evidence on the 4 methods under review to support proposed changes to the 2011 CYPs for current methods and to justify the CYPs proposed for new methods.

To calculate the updated CYP for the Caya diaphragm, we drew upon approaches used for similar products, in this case, previous iterations of the diaphragm to determine the CYP. For Levoplant, which changed in duration of efficacy from 4 to 3 years, we used the past approach to calculate CYP for a 3-year implant. For the hormonal IUD, which changed in duration of efficacy from 5 to 7 years, we maintained the same approach used previously for the hormonal IUD but extended the modeled continuation curve by 2 years. For CYP calculations that account for method effectiveness, we relied on the widely used method-specific effectiveness rates from the *WHO Family Planning - A Global Handbook for Providers*.^8^ Further details on the calculations per method are described in the Results section.

## RESULTS

### Methods That Are New or Becoming Available in Low- and Middle-Income Countries

#### Diaphragm

Diaphragm use has historically been low in LMICs. However, the Caya diaphragm offers several benefits over previous models and presents an opportunity to expand the method mix by increasing method choice. Aside from design improvements to increase comfort and usability, Caya comes in just 1 size, thereby eliminating the need for a fit exam—a key barrier to use in low-resource settings.^6^^,^^9^

Two studies conducted post-2011 were identified in our literature search. In 2015, a randomized controlled trial was conducted with a single-size diaphragm used with a contraceptive gel.^10^ Approximately 54% of participants completed the 6-month study, and the trial found a 10.4% typical use pregnancy probability (at 6 months) that was extrapolated to a 17.8% 12-month probability, which nearly matches the widely used rate (17%) from the WHO Global Handbook.^8^ The only additional data source is a study conducted from 2019–2021 in Niger that estimated a 76.7% 6-month continuation rate.^6^

The 2000 CYP update conducted by Stover et al. assigned 1 CYP to the diaphragm based on how long a woman may typically use it in combination with other factors, such as wastage and discontinuation.^4^ The diaphragm was subsequently removed from the CYP Chart in 2011 because of limited use in USAID-funded programs. Given the desire to add the diaphragm back to the CYP chart and the sparse data post-2011, the updated CYP factor is 1 CYP per diaphragm.

### Methods That Had a Labeled Duration of Use Change

#### Levoplant

Levonorgestrel implants are an effective long-acting reversible contraception option. Levoplant is a levonorgestrel implant that was prequalified by the WHO in 2017^11^ based on a Phase 3 trial conducted in the Dominican Republic.^12^ The predecessor to Levoplant, Sino-Implant (II), was labeled as a 4-year product, and the CYP was initially based on this duration of efficacy. However, based on the trial results that showed markedly higher pregnancy rates in the fourth year,^12^ Levoplant is now labeled as a 3-year product and registered as a 3-year product in all countries where USAID procures the implant.

As the approved duration of use for Levoplant changed from 4 to 3 years, we recalculated its CYP to reflect the duration of efficacy and align with the CYP for the 3-year etonogestrel implant Implanon/ImplanonNXT. The updated CYP is 2.5 per Levoplant implant inserted.

To reflect the change in duration of efficacy from 4 to 3 years for Levoplant, we recalculated its CYP to 2.5 per Levoplant implant inserted.

#### Hormonal IUD

The use of hormonal IUDs as a form of reversible contraception has been limited in LMICs; however, recent pilot studies have shown high user satisfaction and product potential.^13^^–^^19^ This, coupled with product introduction efforts in multiple LMICs, will increase access to and use of the product.^20^ In 2019 and 2020, respectively, the FDA extended the duration of use for the Mirena and Liletta hormonal IUDs from 5 to 7 years. In the coming years, national drug authorities in LMICs will update approvals accordingly. In 2022, the FDA again extended the duration of use for Liletta and Mirena to 8 years; however, this update was made after this current CYP update was completed. Future updates should take this duration of efficacy into account.

In 2011, the CYP calculation for the hormonal IUD with a 5-year duration of use relied on the continuation rates of the Copper-T 380-A IUD and resulted in 3.3 CYP per inserted hormonal IUD. At that time, continuation rate data of the hormonal IUD were limited to U.S.-based clinical trials. Based on data available at that time, there was consensus that the hormonal IUD had continuation rates similar to copper IUDs.^21^ However, since then, data comparing continuation rates of hormonal and copper IUDs have emerged.

The U.S.-based CHOICE study provided robust data comparing the Copper-T 380-A and the hormonal IUD among U.S. FP clients.^22^^–^^24^ These data indicated higher 1- and 2-year continuation rates for the hormonal IUD (88%, 79%, respectively) than for the Copper-T 380-A (84%, 77%, respectively) and equivalent 3-year continuation rates (70% for both methods). Additionally, the continuation rates for the hormonal IUD are nearly equivalent to the continuation rates for contraceptive implants in the 2011 CYP update.

Additional studies conducted in LMICs further support these findings. A prospective cohort study conducted in Zambia and Nigeria showed higher continuation rates for the hormonal IUD (95%) compared to the Copper-T 380-A (89%) in Zambia in the first year and the same continuation rate (87%) for both IUDs in Nigeria.^16^ Two additional studies were considered: a prospective observational study in China^25^ and a 9-country randomized trial with a majority of participants from China (56%).^26^ The prospective observational study had a relatively high 1-year continuation rate for hormonal IUD users (93%)^25^ while the other reported higher continuation rates for the Copper-T 380-A over 1, 3, and 5 years (90%, 80%, 69%, respectively) than the hormonal IUD (84%, 62%, 48%, respectively).^26^ The extent to which study design and copper IUD popularity in China^27^ affected the results is unknown, but they may explain the lower continuation rates for the hormonal IUD in the randomized trial.

Based on this recent evidence, hormonal IUDs in LMICs likely have continuation rates that more closely align with those of hormone-releasing implants and higher continuation rates than the Copper-T-380-A. Because the continuation rates for both IUDs and implants are calculated using the same methodology, just relying on different input data, it was decided to extend the modeled continuation curve for hormone-releasing implants to account for the 7-year duration of efficacy of the hormonal IUDs to determine the CYP, resulting in 4.8 CYPs per device inserted.

The continuation curves created for the 2011 update for IUDs and implants are based on articles that represent real-world usage, with a tendency toward using Demographic and Health Survey data whenever possible. Thus, the focus was on the quality of the data rather than the quantity of articles included in the analysis. For implants, there were 4 articles used in the calculations, 3 representing secondary analysis of Demographic and Health Survey data and 1 study of real-world usage in Senegal.^28^^–^^31^ Data on continuation rates from these articles were used to calculate a curve that is then used to estimate the average duration of use.

The average duration of use was calculated by fitting an exponential decay curve to the continuation data:

$$R={ae}^{-rt}$$

where *R* is retention at time t, *a* is constant that allows for immediate expulsion, *r* is constant that measures the annual rate of discontinuation, and *t* is time expressed in years.

This equation was transformed by taking the natural logarithm of both sides. Then, a regression was run using the log of the continuation rates as the dependent variable and the year as the independent variable. Regression results were transformed back and used to estimate annual retention rates. These annual retention rates were then used to estimate life years of use, which were summed to get the average duration of use.

It is important to remember that with this approach the number of years that are included in the estimates impacts the average duration of use. For example, the same curve was used for estimating continuation for all implants by truncating the data at 3, 4, and 5 years, and now for the 7-year hormonal IUD. Also, as mentioned previously, the effectiveness of the methods is included in the continuation estimates because discontinuations for all reasons, including pregnancy, are looked at together.

### Methods That Had a Presentation Change

#### U.S. Agency for International Development-Supplied Progestin-Only Pills

POPs and combined oral contraceptives (COCs) are both highly effective methods of contraception^8^ supplied by USAID and used worldwide. Before this update, all oral contraceptive pills (POPs and COCs) were provided in 28-pill packs and were both assigned the same CYP–15 cycles, or pill packs, per CYP. USAID currently supplies POPs in packets of 35 pills and no longer provides POPs in 28-day packs. Therefore, it is necessary to differentiate COCs from POPs in the CYP calculation.

To remain consistent with the recommendations from 2011, we eliminated the proportion overlapping from the calculation. To calculate the CYP for POPs, we relied on the following formula:

number required (biological basis)÷effectiveness=CYP

$$\frac{365\;days/year}{35\;pills/pack}÷{93\%\;effectiveness}^{8}=11.18\;cycles/CYP$$

With subsequent rounding up due to suspected wastage, the CYP for POPs is 12 cycles per CYP (0.0833 CYP per pack).

## DISCUSSION

### Standardized Process for Couple-Years of Protection Updates

As contraceptive methods continue to evolve, the CYP indicator will require regular updates to remain current. However, to date, the process for updating the indicator has been ad hoc and lacking standardization. To address this, we have proposed a formal process to both trigger an update and conduct them in the future. Colleagues from Data for Impact have highlighted the importance of standardized indicators, noting that without them, health interventions cannot be compared in an evidence-based fashion, leading to “poor decision making on a large scale, resulting in incorrect assessments and potential investment in less effective programming.”^32^ Global standardization of the CYP indicator will ensure that all programs are measuring this indicator consistently and thus allow for greater comparability and improved decision-making by funders, policymakers, and program implementers. As programmatic data needs change and new evidence is generated, future changes to the standardized process for CYP updates may be needed. However, in the interest of consistency, we recommend following the guidance in this article until any future adjustments are formalized and documented.

Global standardization of the CYP indicator will ensure that all programs are measuring this indicator consistently and will allow for greater comparability and improved decision-making by funders, policymakers, and program implementers.

### Importance and Use of Couple-Years of Protection

CYP is an important indicator for measuring the method-specific coverage of contraceptives provided by FP programs. FP2030 includes CYP as one of the core indicators countries should use to monitor their programs and track trends and progress over time.^33^ It is the only FP2030 core indicator with data that comes directly from routine data systems in countries, such as the health management information system.^33^ However, CYP has limitations, as it cannot be used to estimate actual method use or impact, the number of individuals using a method, whether contraceptive methods are accessible and acceptable to all individuals, and the quality of contraceptive services. Programs should look to other indicators such as contraceptive prevalence rate, discontinuation rate, method information index, and unintended pregnancies to measure such outcomes. When CYP is used in conjunction with other indicators, including the broader set of FP2030 core indicators, a more comprehensive picture of FP programs emerges that enables donors, policymakers, and implementers to make evidence-based decisions to improve programs.

As noted earlier, this update is focused on USAID-procured commodities, though both the CYPs and the process for producing the CYPs are broadly applicable, as the commodities are the same across programs except for the 35-day POP pill pack. Notably, procurement has shifted almost exclusively to the 35-day POP pill pack apart from 2 countries, so this exception is negligible.

## CONCLUSION

Since its inception 50 years ago, CYP has been an integral indicator for FP programs worldwide. As the field of contraceptive research and development continues to generate new evidence on current FP methods and develop new methods, the CYP indicator will need to be updated accordingly. This article not only documents the most recent CYP update but also provides essential guidance to ensure a harmonized and standardized approach to updating CYP.

Using the new CYP factors is required for USAID programs and, in the interest of standard reporting and data-driven decision-making, is strongly recommended for all FP programs. USAID and other international FP stakeholders should regularly monitor FP methods for any changes that require a CYP update and ensure that updates are conducted as needed, thereby ensuring the indicator remains relevant in the future.

## Footnotes

* The U.S. Agency for International Development website states that “the term ‘CYP’ reflects distribution and is a way to estimate coverage and not actual use or impact. The CYP calculation provides an immediate indication of the volume of program activity. CYP can also allow programs to compare the contraceptive coverage provided by different family planning methods.”

## Notes

**Peer Reviewed**

**First Published Online:** April 9, 2024.
